# Sociocultural Appearance Standards and Risk Factors for Eating Disorders in Adolescents and Women of Various Ages

**DOI:** 10.3389/fpsyg.2018.00429

**Published:** 2018-03-29

**Authors:** Bernadetta Izydorczyk, Katarzyna Sitnik-Warchulska

**Affiliations:** Institute of Applied Psychology, Jagiellonian University, Kraków, Poland

**Keywords:** body-image, eating disorders, mass media, girls, women

## Abstract

The main aim of the present study was to verify the level of impact of sociocultural appearance standards (passive awareness and active internalization) have on body dissatisfaction, the desire to engage in a relentless pursuit of thinness, the adoption of a perfectionistic attitude toward the body, and the development of a tendency to engage in bulimic eating behavior, which can develop in adolescent girls and women of varying ages. The study group comprised 234 individuals: 95 secondary school girls, 33 high school girls, 56 female students, and 50 employed women, all of whom were living in southern Poland. Participants were not diagnosed with any psychiatric disorders (including eating disorders). The variables were measured using the Polish version of Garner’s Eating Disorder Inventory and the Polish Sociocultural Attitudes Towards Physical Appearance and Body Image Inventory [based on the SATAQ-3 (Sociocultural Attitudes Towards Appearance Questionnaire Scale-3)]. The findings revealed that the youngest Polish girls (aged 12–15) reported the highest level of risk factors for eating disorders. Among the entire study group, the internalization of appearance standards and the pressure associated with various media messages were determined to be predictors of the pursuit of thinness, regardless of age and body mass index values. The second most significant variable explained by the internalization of sociocultural standards was body dissatisfaction. The internalization of sociocultural norms provided a significant explanation of bulimic tendencies only in the youngest girls. Perfectionism proved not to be affected by the sociocultural impact of mass media. The adult women had the lowest average scores over the entire study population regarding exposure frequency to body images in mass media and regarding the experience of pressure exerted by sociocultural norms. The high level of internalization of sociocultural appearance standards seems to be significantly linked to body satisfaction in women aged 30 and older. Young adolescent girls constitute a high-risk group for a specific psychological proneness to developing eating disorders as a result of the sociocultural influence exerted by mass media. The obtained study results can prove helpful in creating education programs in preventive healthcare aimed particularly at the youngest adolescents.

## Introduction

Previous literature reviews have confirmed the importance of sociocultural messages in highly industrialized countries in regard to strengthening the perception that “thin female bodies” are attractive (Levine et al., 1994; Bearman et al., 2006; Benton and Karazsia, 2015). Such sociocultural standards concerning body image and evaluations of attractiveness are commonly propagated in mass media. In particular, the need for obtaining social approval concerning how one looks and whether one’s body is considered “attractive” is important for adolescent girls and young women in terms of their self-esteem (Clark and Tiggemann, 2008; Izydorczyk and Rybicka-Klimczyk, 2009; McCabe and Ricciardelli, 2009; Cash, 2011; Ferguson K. et al., 2011; Finne et al., 2011; Mond et al., 2011; Gajtkowska, 2013; Józefik, 2014; Izydorczyk, 2015a; Czepczor et al., 2016; Głogowska and Zatorska, 2016).

Many studies have confirmed that women more strongly and regularly feel dissatisfaction with their appearance and, as a result, many intensely pursue the sociocultural ideal of the female body, based on slenderness and thinness, that is promoted in the media (Grabe et al., 2008; Cash, 2011; Ferguson C.J. et al., 2011); it has been shown that such an unrealistic ideal of a slim figure can result in body dissatisfaction and disordered eating (Rodgers et al., 2011). In fact, studies have found that there is a positive correlation between exposure to mass media (TV and magazines) and body dissatisfaction, thin-ideal internalization, and disordered eating (Thomsen et al., 2002; Levine and Murnen, 2009). The influence of the media has a key impact on appearance satisfaction in terms of thinness and fatness (Levine and Murnen, 2009). Levine and Smolak (2006) indicated that women’s dissatisfaction with their bodies is a derivative of three components: the idealization of slenderness and leanness, an irrational fear of fat, and a conviction that weight and shape are central determinants of one’s identity.

Factors such as body-image dissatisfaction, the restrictive pursuit of thinness, the adoption of a perfectionistic attitude toward the body, and the development of bulimic tendencies are often indicated in scientific research as predictors of eating disorders (Stice et al., 1994, 2013; Tiggemann, 2003; Garner, 2004; Jones and Crawford, 2006; Żechowski, 2008; Striegel-Moore et al., 2009; Izydorczyk, 2015b). However, most researchers focus on selected risk factor. The clinical experience of the authors indicates that the risk factors of eating disorders should not be considered separately. These factors seem to constitute the specific syndrome, related to the culture of thinness.

It would be a great simplification to say that mass media has a direct impact on the occurrence of eating disorders, and research results are ambiguous in this respect (Stice et al., 1994, 1998; Tiggemann, 2003). Rodgers (2016) proposed a theoretical model that suggests the existence of two possible mechanisms behind the relationship between promoted appearance standards and behaviors that are considered risk factors for eating disorders. The first mechanism is based on the assumption that sociocultural discourse on healthy weight promotes the internalization of anti-fat attitudes (in regard to oneself and toward others) and the need to control weight. The second mechanism concerns the formation of the belief that lifestyle factors (such as diet or physical activity) play an important role in weight control. As a consequence, this can lead to a preoccupation with weight, which is then followed by disordered eating and excessive exercise.

Models of media influence argue that normative beliefs concerning society, such as those shown in media, become attitudes about the self (Levine and Smolak, 1996; Levine and Murnen, 2009). For example, Levine and Murnen (2009), in their critical review, indicated that mass media should be treated as a variable risk factor that might later become a causal risk factor. In their meta-analysis, they indicate that when considering the relationship between the media and eating disorders, variables such as awareness of the importance in society of a thin ideal, the internalization of this ideal, and perceived pressure from the media to be thin should be examined.

It is also worth highlighting that contemporary research has applied a large variety of methods to measure the variables presented above. One valid measure for these variables is the *Sociocultural Attitudes Towards Appearance Questionnaire* (SATAQ; Knauss et al., 2009; Sánchez-Carracedo et al., 2012). However, many researchers use this scale mainly to study the relationship between sociocultural appearance standards, promoted in the media, and selected risk factors concerning eating disorders (e.g., body dissatisfaction; Stice and Whitenton, 2002). Definitions of these factors also differ; some researchers use the *Eating Disorders Inventory* (EDI) in their research, but this is not a standard. Moreover, there is a lack of research on the relationships between sociocultural standards of appearance and the entire team of risk factors involved in eating disorders.

Efforts to identify sociocultural predictors that contribute to the development of body-image distortion as a result of eating disorders are not very common in studies conducted on adult women (aged 30 and older) when compared to studies on adolescents. Further, as mentioned above, there is also a lack of studies focusing on broad populations of women (young and adult) with the goal of measuring the differences between them and the strength of the correlations between factors commonly considered risk factors for eating disorders.

There is also doubt concerning whether the sociocultural standards of appearance are universal. The culture of thinness is common in Western Europe, the United States, and more often on the other continents. Nielson et al. (2013) indicate that exposure to Western models in the media has likely contributed to a rejection of stereotypical Asian facial features. The European findings show that young people from the United Kingdom seem to demonstrate the most intense internalization of the thin ideal and pressure from the media to look a certain way (Argyrides, 2013). Swami et al. (2010) found that the heavier bodies may be preferred in low-socioeconomic-status sites compared to high-socioeconomic-status sites in Malaysia and South Africa, but not in Austria.

However, the cross-cultural explorations suggest that overall differences are limited and mainly associated with differences in socioeconomic development (Swami et al., 2010). Considering the above, it seems important to analyze girls and women of different ages and from the same sociocultural area. Further, in light of the findings of numerous studies concerning the impact of sociocultural standards in Western, highly developed countries, it appears that conducting such research in dynamically developing countries in Central and Eastern Europe would be valuable. Moreover, this type of research can also support meta-analyses, taking into account different populations.

The authors’ research for this article comprised a project that was performed over several years, which concerned searching for sociocultural risk factors for eating disorders among groups of Polish girls and women. Poland is a dynamically developed country. In the conducted research, the authors decided to use SATAQ Scale-3 (SATAQ-3) to measure sociocultural appearance standards and EDI to measure variables recognized in the literature as risk factors for eating disorders (such as dissatisfaction with the body, the restrictive pursuit of slimness, and bulimic tendencies). The feature of perfectionism (measured by EDI) was also included in the scope of variables, because of its importance for the development and course of eating disorders.

The authors assumed that mass media are a major part of the lives of many children, adolescents, and adults. The assimilation of sociocultural appearance standards, promoted in the media, is of key importance. This idea is supported by research showing that it is not just the frequent exposure to the media, but also referring to media content to oneself that leads to an assessment of one’s own body (Wilcox and Laird, 2000). Therefore, it can be concluded that this process requires a degree of awareness and is dependent on intrapsychic processes. For this reason the authors decided to focus on the analysis of the self-perception, individual attitude to media consumption and individual assessment of media impact. These factors are little understood. The main aim of the present study was to empirically verify the level of impact sociocultural appearance standards (awareness and active internalization) have on body dissatisfaction, the desire to engage in a relentless pursuit of thinness, the adoption of a perfectionistic attitude toward the body, and the development of a tendency to engage in bulimic eating behavior (self-inducing vomiting and using other purgative behaviors to eliminate food from the stomach), which can develop in adolescent girls and women of varying ages.

Specifically, the research objective was to seek answers to the following research questions:

(1) Are sociocultural standards of appearance related to the development of risk factors for eating disorders (dissatisfaction with the body, restrictive pursuit of slimness, bulimic tendencies, and perfectionism) in modern-day girls and women?

(2) Is the relationship between sociocultural standards of appearance and risk factors of eating disorders specific to the developmental period (early adolescence, middle and late adolescence, young adulthood, middle adulthood)?

## Materials and Methods

### Procedure

This study was conducted between 2012 and 2016 in southern Poland. The authors planned to examine several hundred women, and it was decided to take into account developmental differences over various stages of life (second research question). For this reason, four age-based subgroups were distinguished: 12–15 (early adolescence), 16–20 (middle and late adolescence), 21–29 (young adulthood), and women over 30 (middle adulthood). The applied divisions into subgroups corresponds to the stages of development indicated in developmental psychology, and takes into account the age ranges of the girls and women who were examined in related research.

The categorizations for the research subgroup were based on a clinical interview [questions concerning age, previous and required treatment of eating disorders, mental disorders, and body mass index (BMI)]. Due to the purpose of the research (examination of risk factors, not symptoms of eating disorders), the study only included persons without mental disorders; specifically, the exclusion criteria were: disability and related visible deformities of the body; previous diagnosis of anorexia, bulimia, or other types of eating disorders; and mental disorders associated with the development of a distorted body image (psychotic, dysmorphia).

Based on literary sources, research findings, and the authors’ clinical observations, the following two main variables were defined in the study design:

(1) Risk factors for eating disorders: a criterion (dependent) variable that comprises four elements:

- Body dissatisfaction: a variable describing the severity of a negative emotional attitude toward one’s own body, its shape, and measurements.
- Pursuit of thinness: a variable describing the severity of preoccupation with the pursuit of thinness, the desire to have a thinner body, and increased anxiety and concerns regarding gaining weight.
- Bulimic tendencies: a variable describing a specific cognitive disposition related to a lack of control of eating behavior that defines the severity of obsessive thoughts concerning eating, binge eating, and provoking various compensatory reactions of the body (self-induced vomiting, using laxatives without any medical justification).
- Perfectionism: a variable describing an individual’s tendency to form excessively high expectations of himself/herself and seek to fulfill the highest possible standards in terms of personal achievements and activities in life, leading him/her to engage in excessive self-criticism of his/her body, successes, and achievements.

(2) Sociocultural appearance standards: a predictor (independent) variable, defined by a person’s level of adaptation to sociocultural standards of body appearance promoted in the media (audio and visual media messages concerning appearance). This variable comprises three elements:

- Internalization: a variable describing the assimilation level (strength) of norms concerning appearance created by modern culture and mass media (TV, radio, magazines and newspapers, advertisements, etc.).
- Pressure: a variable describing the level (strength) of the pressure experienced from various media messages (TV, radio, magazines and newspapers, advertisements, etc.) promoting body-image standards.
- Exposure to body images promoted in mass media: a variable describing the frequency of consumption of various information promoted in mass media pertaining to body-image standards and norms of appearance.

An additional control variable used in the study was BMI, which comprises a value derived from the body mass divided by the square of the body height in meters (Menzel et al., 2011). On this index, optimal weight is assumed to range from 19.5 to 24.5, with values below the average indicating underweight or a pathological loss of weight, and values above indicating overweight.

### Participants

The study involved initially 250 girls and women, 16 of whom were excluded, after the clinical interview. There were six girls aged 12–15 treated for anorexia, five girls aged 16–20 treated for anorexia and binge eating disorder, and five women over 30 treated for chronic depression.

The study group finally comprised 234 individuals: secondary school girls, high school girls, female students (full-time and part-time students attending courses in the fields of humanities, social studies, and biological science), and employed women, all of whom were living in southern Poland. The research group was homogeneous in terms of sociodemographic conditions (place of residence, age, gender). The following subgroups were created:

Subgroup I (95 adolescent girls aged 12–15; these represented early adolescence; mean age = 14.0, mean BMI = 19.0)
Subgroup II (33 adolescent girls aged 16–20; these represented middle and late adolescence; mean age = 18.2, mean BMI = 20.0)
Subgroup III (56 women aged 21–29; these represented young adulthood; mean age = 25.5; mean BMI = 23.0)
Subgroup IV (50 women aged 30 and older; these represented middle adulthood; mean age = 35.5, mean BMI = 24.4)

The criteria applied when selecting participants for these subgroups included: participants’ ages (12–50 years), BMIs (optimal BMI values at a given age), and the absence of any eating disorders (e.g., anorexia nervosa and bulimia nervosa) that required treatment. The BMI values of all the adolescent girls and women participating in the study were within normal limits and corresponded to the optimal weight (18.5–24.9).

### Compliance With Ethical Standards

This study was conducted in accordance with the recommendations of an institutional research committee (Research Ethics Committee of the Institute of Applied Psychology, Jagiellonian University, Krakow) and was determined to conform with the 1964 Helsinki declaration and its later amendments or comparable ethical standards, as written informed consent was obtained from all subjects. Informed consent was also obtained from the parents or legal guardians for all participants under the age of 16.

The protocol was approved by the Research Ethics Committee of the Institute of Applied Psychology, Jagiellonian University, Krakow.

### Instruments

The clinical interview was used to select study participants. The interview questions concerned the objective indicators, such as age, body weight, and height (to estimate the BMI), the presence of disabilities and related visible deformities of the body, previous diagnosis of eating disorders (anorexia, bulimia, or other types of eating disorders), and mental disorders associated with the development of distorted body image (body dysmorphic disorder, psychotic disorders, personality disorders, depression, bipolar disorders).

The predictor variable, and its components, were measured using the Polish Sociocultural Attitudes Towards Physical Appearance and Body Image Inventory (SATPABI), which was developed by Izydorczyk (2014). It is based on the SATAQ-3 developed by Thompson et al. (2004); the SATAQ-3 is a measure of the internalization of appearance ideals and appearance pressures. To create the SATPABI, a 24-item Polish-language questionnaire was drafted based on a double back-translation of the English version of the SATAQ-3. The said questionnaire was used in a pilot study on a population of 140 Polish females who did not manifest traits of eating disorders. The items employed in the pilot study described basic manifestations of assimilated sociocultural appearance standards regarding the following variables: internalization, pressure, and exposure to body images promoted in mass media. The obtained pilot study results were then subjected to a reliability analysis, in which sampling adequacy was estimated at KMO = 0.983. Bartlett’s test results allowed the authors to disprove the identity matrix hypothesis (χ^2^ = 3046.280; *df* = 276; *p* < 000). Further, in order to verify and estimate statistical indicators for all positions in the questionnaire, a factor analysis was conducted and the identified factors were subjected to varimax rotation with Kaiser normalization.

The SATPABI, like the SATAQ-3, has three subscales, assessing internalization (general, athlete; nine items), pressure (seven items), and exposure to body images promoted in mass media (eight items); each item on each scale is given between one and five points. For each scale, the higher the sum of points, the greater the severity of the indicator.

The criterion variable and its components were measured using the Polish version of Garner’s EDI, which was developed by Żechowski (Garner, 2004; Żechowski, 2008) (Psychological Assessment Resources granted permission for one of the authors to conduct research using the EDI). The total scores for the dissatisfaction with the body, drive for thinness, bulimia, and perfectionism scales in the EDI questionnaire were used as indicators of risk factors for eating disorders. For each scale, the higher the sum of the points (body dissatisfaction: nine items, pursuit of thinness: seven items, bulimic tendencies: seven items, and perfectionism: six items; each item of each scale is scored between zero and three points), the greater the severity of the indicator.

### Statistical Methods

The examined subgroups differed in terms of the number of members, but the size of each subgroup was sufficient to conduct the planned statistical analyses.

The first stage of the statistical analysis contained measurements of mean values relating to the severity of each variable (perfectionism, body dissatisfaction, leanness, bulimic tendencies), and mean values (*M*) and standard deviations (*SD*) concerning the intensification of explanatory variable factors: internalization, pressure of sociocultural norms, and searching for information about body image in mass media. The significance was measured using one-way analysis of variance (ANOVA) with the least significant difference (LSD) test applied *post hoc*.

Further, a stepwise regression analysis was applied to determine the extent to which sociocultural appearance standards explain the development of excessive body dissatisfaction, the pathological and relentless pursuit of thinness, bulimic tendencies, and perfectionism in the studied subgroups of girls and women.

## Results

### Characteristics of the Dependent Variable in the Participants

**FIGURE 1:**
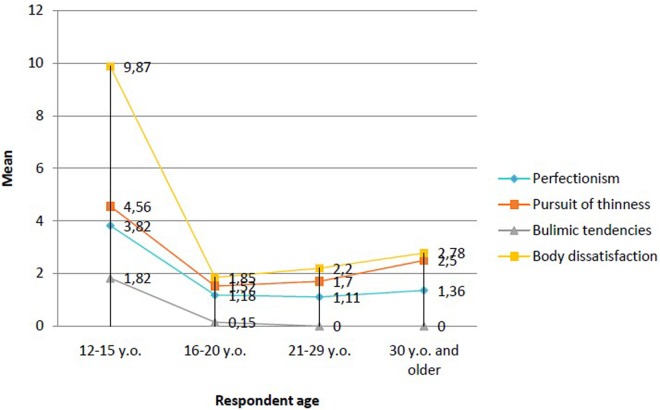
Statistics concerning the differences between the subgroups regarding the severity of risk of developing eating disorders.

**Figure 1** shows the statistically significant differences between the various subgroups in terms of the average severity of “risk factors for eating disorders.” Here, it can be seen that, compared to the older adolescents and women participating in this study, subgroup I showed the highest scores for body dissatisfaction, along with the highest scores for relentless pursuit of thinness and perfectionism. Further, the verified constituents of the dependent variable (body dissatisfaction, pursuit of thinness, bulimic tendencies, perfectionism) proved to have the strongest effect on the youngest adolescent girls when compared to the other participants (*p* < 0.001 for the difference between subgroup I and subgroups II, III, and IV; **Table 1**). Meanwhile, compared to the severity of the other aforementioned variables, the severity of bulimic tendencies was lowest among the youngest adolescents. However, the youngest girls obtained much higher values on the scale of perfectionism than the subjects from the other subgroups (**Figure 1**). Moreover, this high score for perfectionism represents a significant trait that differentiates subgroup I from all of the other females, who scored equally on average (**Figure 1** and **Table 1**).

**Table 1:** Descriptive statistics of the criterion and predictor variables (*n* = 234).

Age (group)	Perfectionism	Pursuit of thinness	Bulimic tendencies	Body dissatisfaction	Internalization of sociocultural norms	Exposure to body image in mass media	Pressure of sociocultural norms

	*M* (*SD*)	*M* (*SD*)	*M* (*SD*)	*M* (*SD*)	*M* (*SD*)	*M* (*SD*)	*M* (*SD*)
12–15 (I)	3.82 (3.76)	4.56 (4.98)	1.82 (3.87)	9.87 (7.84)	23.80 (7.773)	23.76 (6.253)	17.61 (5.23)
16–20 (II)	1.18 (0.39)	1.52 (0.51)	0.15 (0.36)	1.85 (0.36)	19.91 (3.574)	25.30 (4.081)	19.61 (4.19)
21–29 (III)	1.11 (0.31)	1.70 (0.46)	0.00 (0.00)	2.20 (0.77)	22.82 (4.577)	25.11 (5.376)	17.70 (5.40)
30 and older (IV)	1.36 (0.48)	2.50 (0.51)	0.00 (0.00)	2.78 (0.42)	22.08 (3.300)	24.54 (3.945)	16.04 (4.01)
ANOVA	*F*(3,230) = 21,79^∗∗∗^	*F*(3,230) = 13,33^∗∗∗^	*F*(3,230) = 9,78^∗∗∗^	*F*(3,230) = 42,60^∗∗∗^	*F*(3,230) = 3,86^∗∗^	*F*(3,230) = 1,10^∗∗^	*F*(3,230) = 3,53^∗∗^
*Post hoc* (*LSD*)*p*-value	<0.001 Group I > 2,3,4	<0.001 Group I > 2,3,4	<0.001 Group I > 2,3,4	<0.001 Group I > 2,3,4	<0.010 Group I > II, and group II < III	Non-significant	<0.016 Group I < II, and group II > IV


*M, mean; SD, standard deviation. ^∗∗^*p* < 0.01, ^∗∗∗^*p* < 0.001.*

### Characteristics of the Independent Variable in the Participants

**FIGURE 2:**
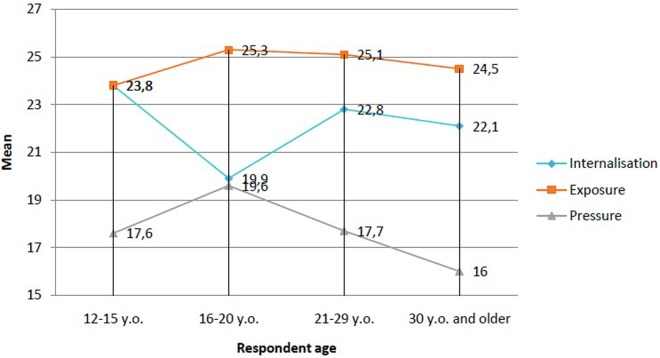
Statistics concerning the differences between the subgroups regarding the severity of sociocultural appearance standards.

The results also implied that the greatest significant difference in regard to the predictor variable was between subgroup I and all other participants (**Figure 2**). Detailed analysis indicated that this effect was mainly due to differences in average values for the internalization and pressure variables (**Table 1** and **Figure 2**). The youngest girls showed much higher values on the scale of internalization, especially in relation to late adolescents. Girls aged 16–20 showed the lowest level of assimilation of sociocultural appearance standards. Concurrently, the analyses indicated that the youngest girls experienced less pressure from various media messages than girls in late adolescence. Meanwhile, women in middle adulthood showed the lowest level of sociocultural pressure. The variable “exposure to body images promoted in mass media” did not differ between groups.

### Characteristics of the Sociocultural Predictors of Eating Disorders

Statistical analyses and regression models for the tested variables showed that sociocultural appearance standards have a varied impact on the development of the risk factors for eating disorders. Based on the obtained adjusted *R*^2^ and beta (β) coefficient values, presented in **Table 2**, one can conclude that only seven models for the sociocultural appearance standards variable explain the significant impact internalization and pressure have on selected constituents of the variables for risk factors for eating disorders (body dissatisfaction, pursuit of thinness, bulimic tendencies). Together with the Fisher test results and coefficients estimated based on the said results (at *p* < 0.001), the models presented in **Table 2** were found to be statistically significant.

**Table 2:** Summary of sociocultural tested predictors.

Dependent variable	Aged 12–15 (*n* = 95)	Aged 16–20 (*n* = 33)	Aged 21–29 (*n* = 56)	Aged 30 and older (*n* = 50)
				
Perfectionism	in^∗^	in^∗^	in^∗^	in^∗^
Pursuit of thinness	Adjusted *R*^2^ = 0.299	Adjusted *R*^2^ = 0.167	Adjusted *R*^2^ = 0.084	Adjusted *R*^2^ = 0.415
	*F*(3,91) = 14.36^∗∗∗^	*F*(1,31) = 7.44^∗∗^	*F*(1,54) = 6.08^∗∗^	*F*(2,47) = 18.38^∗∗∗^
Internalization of sociocultural norms	β = 0.315^∗∗^	β = 0.440^∗∗^	4	β = -0.533^∗∗∗^
Pressure of sociocultural norms	β = 0.402^∗∗∗^	–	β = 0.318^∗∗^	β = -0.240^∗^
Bulimic tendencies	Adjusted *R*^2^ = 0,127 *F*(1,91) = 14.63^∗∗∗^	in^∗^	in^∗^	in^∗^
Internalization of sociocultural norms	β = 0.369^∗∗∗^			
Body dissatisfaction	Adjusted *R*^2^ = 0.239 *F*(1,91) = 30.64^∗∗∗^	in^∗^	in^∗^	Adjusted *R*^2^ = 0.088 *F*(1,48) = 5.74^∗^
Internalization of sociocultural norms	β = 0.498^∗∗∗^			β = 0.327^∗^


*in, insignificant. ^∗^*p* < 0.05, ^∗∗^*p* < 0.01, ^∗∗∗^*p* < 0.001.*

In regard to summarizing the significant regression models, one ought to note that, in subgroup I, variables associated with the various risk factors for eating disorders (body dissatisfaction and pursuit of thinness) were most strongly affected by sociocultural appearance standards. Further, among the verified constituents of the sociocultural appearance standards variable, internalization (in groups I, II, IV) and pressure (in groups I, III, IV) proved to be significant.

Among the entire study group, sociocultural appearance standards were determined to be predictors of the pursuit of thinness, regardless of age and BMI values. The obtained values suggest that the pursuit of thinness variable is positively correlated with the pressure associated with various media messages (in girls aged 12–15 and women aged 21–29) and the internalization of appearance standards (in girls aged 12–15 and those aged 16–20). Moreover, the conducted analysis indicated that women in middle adulthood engage in a stronger pursuit of thinness, provided they have less internalized sociocultural appearance standards and experience less sociocultural pressure (trend).

The second most significant variable explained by the internalization of sociocultural standards is body dissatisfaction. The regression analysis conducted for the body dissatisfaction variable in subgroup I and subgroup IV revealed high levels of assimilation of appearance standards (internalization) to be a significant predictor.

In the case of bulimic tendencies, only internalization of sociocultural norms provided a significant explanation of this tendency in subgroup I. In the other subgroups, the sociocultural impact did not provide a significant explanation for bulimic tendencies.

No significant forward stepwise regression models were identified in the measurement of the impact exerted by the sociocultural appearance standards on the development of perfectionism among the participants.

## Discussion

The present research proves the existence of a relationship between the occurrence of risk factors for eating disorders (body dissatisfaction, pursuit of thinness, bulimic tendencies) and the sociocultural appearance standards (internalization and pressure), promoted in mass media, in the studied girls and women. The results of conducted research also indicate that the relationship between sociocultural standards of the appearance and risk factors of eating disorders is specific to the developmental period.

In particular, the findings revealed that the youngest Polish girls reported the highest level of risk factors for eating disorders. Girls aged 12–15 were found to be the least satisfied with their own bodies; meanwhile, compared to older adolescents and young and mature women, the youngest participants also reported significantly higher levels of efforts to become thin, bulimic tendencies, and perfectionism. These results concerning the young adolescent girls support research conducted by other authors (Stice et al., 2008; Żechowski, 2008; Izydorczyk and Rybicka-Klimczyk, 2009; Striegel-Moore et al., 2009; Brytek-Matera and Rybicka-Klimczyk, 2012; McCabe et al., 2012; Izydorczyk, 2015a,b); most of these previous studies focused on body dissatisfaction and the pursuit of thinness. Meanwhile, Ata et al. (2007) indicated that as many as 70% of adolescent girls would like to have a smaller body size. On a related note, many studies have reported that dissatisfaction with appearance is more prevalent among adolescent girls than adolescent boys (Neumark-Sztainer et al., 2002; McCabe and Ricciardelli, 2004; Ata et al., 2007; Xu et al., 2010; Lawler and Nixon, 2011; McCabe et al., 2012); girls seem to be more interested in losing weight, in contrast to boys, who have a greater desire to increase muscle (Mellor et al., 2009).

The body dissatisfaction and pathological pursuit of thinness identified among the youngest adolescents might be motivated by psychosexual development processes and self-esteem based on the principle “I am what I look like.” Furthermore, the said life stage is characterized by body-image evaluation (particularly among peers, but also parents) adopting an increased role in social environments. McCabe and Ricciardelli (2003) indicated the most significant influences for these factors; however, clinical observations have indicated that mass media is becoming an increasingly important part of the lives of today’s children and adolescents (Comstock and Scharrer, 2007; Hayes and Tantleff-Dunn, 2010; Lamb and Peterson, 2012). According to Gajtkowska (2013), in teenagers’ opinions, the media are currently the most common source of information about appearance (78.67%).

However, Field et al. (2001), in an examination of a group of over 6,900 girls aged 9–15 years, obtained contrasting results. Here, their analyses did not clearly show that there was a correlation between the media (frequency of reading women’s fashion magazines, use of diets to achieve a weight consistent with common standards) and dissatisfaction with appearance. Thus, it seems that exposure to mass media is not a sufficient explanatory factor; the assimilation of sociocultural standards may also play a key role. This hypothesis is confirmed by the results of the present research.

The regression analysis of the body-image dissatisfaction variable for subgroup I revealed the internalization of appearance standards to be a significant predictor in this regard. The greater the body-image dissatisfaction among the youngest adolescent girls, the greater the internalization of sociocultural norms regarding body-image standards. It is interesting to note that internalization also proved to be the only predictor of bulimic tendencies, and only for subgroup I. Pressure and the internalization of sociocultural appearance standards were also found to be significantly positively correlated with the pursuit of thinness in this subgroup. Supporting this finding, Botta (2003), Shroff and Thompson (2006), and Mäkinen et al. (2012) all found a similar dependence between the internalization of sociocultural norms and body dissatisfaction among the youngest girls. This could be explained by the fact that, at the beginning of adolescence, girls are significantly affected by biological and psychosocial processes. The age of 12–15 is a developmental period, the purpose of which is to determine one’s own identity. During this period, it is particularly important to define one’s self and reconcile the private self and the public self. The youngest adolescents are influenced by society to adjust to the new tasks they must fulfill while concurrently playing their psychosocial roles as teenagers (a daughter, a student, a girlfriend, a colleague, a friend). Consequently, at this point in their development, it is possible that they only develop their psychological reactions to teenage angst, and do not endeavor to negate the presence of image-related pressure exerted by social norms.

It was observed that the youngest and older adolescents showed significantly different levels of pressure in terms of sociocultural norms regarding the binding standards of appearance. In the analysis of the results for subgroup II, the level of internalization of sociocultural norms was found to be significantly lower than that for all other subgroups. These results correspond with the higher levels of pressure experienced by these participants as a result of sociocultural norms. It is worth considering whether adolescents in the so-called “teenage angst” stage (16–20 years) do not manifest a general negation of the impact the environment can have on their behavior, which would be reflected in their decisions to negate the social pressure experienced.

Furthermore, subgroup I showed a level of pressure as a result of sociocultural norms that was similar to that experienced by subgroup III. The higher the level of pressure, the greater the body dissatisfaction. It is worth noting that young women of this age are usually beginning to fulfill numerous new psychosocial roles that are imposed on them (university student, employee, romantic partner, wife, mother, etc.); therefore, they can feel similar social pressure to the youngest girls. Adolescents and young women react in a particularly strong manner as a result of the psychological conflicts experienced in their life stages (identity crises, psychological separation, and individualization contrasted by a strong need for social acceptance and self-esteem; Clark and Tiggemann, 2008; Izydorczyk and Rybicka-Klimczyk, 2009; Ferguson K. et al., 2011; Mond et al., 2011; Izydorczyk, 2015a).

Halliwell (2013) suggests that showing appreciation to young women protects them from negative environmental appearance messages. On the other hand, Bair et al. (2012) indicated that young adults use the Internet as their primary news source and, for this reason, they are more likely to internalize appearance standards; as a consequence, they are at a greater risk of engaging in disordered eating (Bair et al., 2012).

The results obtained from women aged 30 and above are particularly interesting. There is a lack of research on body image and desire for thinness among adult women. Bulimic tendencies among the adult participants (aged 30 and older) proved to be insignificant; adult women also had the lowest average scores over the entire study population regarding exposure frequency to body images in mass media and regarding the experience of pressure exerted by sociocultural norms. The regression analysis also indicated an interesting correlation: the more intensified the internalization of sociocultural appearance standards, the less thinness is pursued among women aged 30 and older. A similar trend can be observed between pursuit of thinness and pressure from sociocultural appearance standards. These results are surprising, especially because previous studies have indicated a reverse relationship in this regard (Dittmar and Howard, 2004; Kilpela et al., 2015).

Kilpela et al. (2015) noticed that body image may be more complex for adult women than for younger women. Aging-related physiological changes shift the female body further away from the thin-young ideal; further, life priorities and psychological factors also evolve with age. Pregnancy, employment, household obligations, and child care are factors that can protect self-image (see Kilpela et al., 2015). Additionally, according to Fredrickson and Roberts (1997), middle-aged women may experience decreased objectification as they age. These results could be also explained by the fact that the life stage of over 30 years is characterized by higher psychological and social maturity. The women of this age in the present study underwent upbringing and performed socialization for many years under the cultural conditions of a highly industrialized country, Poland. Given the fact that their personalities were well structured and that they reported no mental disorders, it can be stated that mature women are capable of opposing the excessive sociocultural influences that promotes unhealthy behavior, e.g., an overly thin body image. On the other hand, the oldest of the participants were born and grew up in times when access to the media was not so common. Perhaps, they have not assimilated the sociocultural standards of appearance promoted in contemporary media.

Perfectionism proved not to be affected by the sociocultural impact of mass media. Bardone-Cone et al. (2017) highlighted maladaptive perfectionism as a complex structure related to both disordered eating and anxiety; meanwhile, Petersson et al. (2017) claimed that it is important to investigate patients’ definitions of perfectionism, which the psychometric measures do not reflect.

The present study was performed on a Polish population of girls and women. The results of the SATAQ-3 scale obtained in our study seem to be slightly lower in terms of internalization and exposure, compared to the results achieved by respondents from other European countries (Argyrides, 2013). This observationshould be treated with great caution. SATAQ scales, used in different countries, have different versions and validations.

However, observations in other countries indicate that individuals who invest more time in their appearance, who have internalized the thin ideal, who feel pressured by the media to look a certain way, and who consider the media as a good source of information for body-image issues are significantly more likely to develop disordered eating behavior and patterns (Argyrides, 2013). The results conform with those of a study that surveyed 7,434 individuals in 10 major world regions in regard to body-weight ideals and body dissatisfaction, which indicated that body dissatisfaction is connected to Western media exposure (Swami et al., 2010). Our study also supports a previous study featuring a cross-western model, which contained a similar body ideal (Rodgers et al., 2011); such an internalized appearance standard can lead to body dissatisfaction and strengthen the pursuit of thinness and disordered eating. Further, the interesting results we obtained concerning adult women may also reflect the cultural roles of women in southern Poland; however, this issue requires further research. These findings also have important implications for those seeking to prevent eating disorders through the implementation of measures that are tailored to individual age groups.

### Limitations and Future Directions

The conducted study is characterized by some limitations pertaining to both the sampling method and the research procedure. Firstly, the study participants (despite being selected in accordance with the research objective and the required procedure) might have constituted a specific sample of adolescent girls and women with a specific background, and consequently the conclusions obtained from the study findings are likely not to be applicable to the remaining population. Nevertheless, the use of a common socio-demographic criterion for sampling and the number of participants support the validity of the conducted study. Secondly, in order to investigate the dynamics of the psychological processes and motivations that drive body-focused behavior, particularly concerning factors that generate body-image distortions typical of eating disorders, longitudinal studies are required, as these would ensure higher reliability and accuracy in the evaluation of the research material and would also allow results to be compared either over several years or over a brief period, when the participants are contacted by the researchers. In fact, a long-term longitudinal study on a group of females would ensure a more in-depth measurement of the processes underlying the development of negative attitudes toward the body. However, considering the time-consuming nature of such studies and the low possibility that such studies can be conducted, as a result of difficulties accessing study groups and developing appropriate research procedures, this form of procedure was not pursued in the present study. Finally, given the specified research objectives and the procedure, as well as the measurement methods presented in the literature, it seems likely that the adopted assumption and research procedure can be implemented by means of transversal studies.

## Conclusion

Young adolescent girls constitute a high-risk group in regard to possessing a specific psychological proneness to developing eating disorders as a result of the sociocultural influence exerted by mass media. Two main sociocultural predictors (internalization and pressure of sociocultural norms) play a significant role in explaining the development of body dissatisfaction and the pathological pursuit of thinness among Polish adolescent girls and women. In relation to perfectionism, no significant role of internalization and sociocultural pressure was observed. Meanwhile, in women aged 30 and over, the high level of internalization of sociocultural appearance standards seems to be significantly linked to body satisfaction. In the future, intercultural research would be interesting, especially studies focusing on women in middle adulthood. The obtained study results can prove helpful for creating education programs in preventive healthcare aimed particularly at the youngest adolescents.

## Author Contributions

BI made substantial contributions to the conception of the work and to the interpretation of data. BI and KS-W made substantial contributions to the design of the work and the interpretation of data and to drafting the work and revising it critically for important intellectual content. Both authors gave final approval of the version to be published and agreed to be accountable for all aspects of the work.

## Conflict of Interest Statement

The authors declare that the research was conducted in the absence of any commercial or financial relationships that could be construed as a potential conflict of interest.

## References

[B1] Argyrides M. (2013). *On the Cover: Media Influences, Body Image and Disordered Eating in European Youth.* Brussels: European Confederation of Youth Clubs.

[B2] Ata R. N. Ludden A. B. Lally M. M. (2007). The effect of garden and family, friend and media influences on eatingbehaviors and body image duringadolescence. *J. Youth Adolesc.* 36 1024–1037. 10.1007/s10964-006-9159-x

[B3] Bair C. E. Kelly N. R. Serdar K. L. Mazzeo S. E. (2012). Does the Internet function like magazines? An exploration of image-focused media, eating pathology, and body dissatisfaction. *Eat. Behav.* 13 398–401. 10.1016/j.eatbeh.2012.06.003 23121797

[B4] Bardone-Cone A. M. Lin S. L. Butler R. M. (2017). Perfectionism and contingent self-worth in relation to disordered eating and anxiety. *Behav. Ther.* 48 380–390. 10.1016/j.beth.2016.05.006 28390500

[B5] Bearman S. K. Presnell K. Martinez E. (2006). The skinny on body dissatisfaction: a longitudinalstudy of adolescent girls and boys. *J. Youth Adolesc.* 35 217–229. 10.1007/s10964-005-9010-9 16912810 PMC1540456

[B6] Benton C. Karazsia B. T. (2015). The effect of thin and muscular images on women’s body satisfaction. *Body Image* 13 22–27. 10.1016/j.bodyim.2014.11.001 25528369

[B7] Botta R. A. (2003). For your health? The relationship between magazine reading and adolescents’ body image and eating disturbances. *Sex Roles* 48 389–399. 10.1023/A:1023570326812

[B8] Brytek-Matera A. Rybicka-Klimczyk A. (2012). Ocena nasilenia objawów syndromu gotowości anorektycznej u młodych kobiet - badania pilotażowe [Assessment of anorexia readiness syndrome escalating symptomsin young women – a pilot study]. *Stud. Psychol.* 12 23–26.

[B9] Cash T. F. (2011). “Crucial considerations in the assessment of body image,” in *Body Image: A Handbook of Science, Practice, and Prevention*, eds Cash T. F. Smolak L. (New York, NY: Guilford Press), 129–137.

[B10] Clark L. Tiggemann M. (2008). Sociocultural and individual psychological predictors of body image in young girls: a prospective study. *Dev. Psychol.* 44 1124–1134. 10.1037/0012-1649.44.4.1124 18605839

[B11] Comstock G. Scharrer E. (2007). *Media and the American Child.* Burlington, MA: Academic Press.

[B12] Czepczor K. Kościcka K. Brytek-Matera A. (2016). Społeczno-kulturowe postawy wobec własnego wyglądu i niezadowolenie z ciała u kobiet i mȩżczyzn w okresie późnej adolescencji: badania wstêpne [The sociocultural attitudes towards appearance and body dissatisfaction among late adolescence: a pilot study]. *Pol. Forum Psychol.* 21 364–377. 10.14656/PFP20160303

[B13] Dittmar H. Howard S. (2004). Thin-ideal internalization and social comparison tendency as moderators of media models’ impance on women’s body-focused anxiety. *J. Soc. Clin. Psychol.* 23 768–791. 10.1521/jscp.23.6.768.54799

[B14] Ferguson K. Munoz M. E. Contreras S. Velasquez K. (2011). Mirror, mirror on the wall: peer competition, television influences, and body image dissatisfaction. *J. Soc. Clin. Psychol.* 30 458–483. 10.1521/jscp.2011.30.5.458

[B15] Ferguson C. J. Winegard B. Winegard Bo M. (2011). Who is the fairest one of all? How evolution guides peer and media influences on female body dissatisfaction. *Rev. Gen. Psychol.* 15 11–28. 10.1037/a0022607

[B16] Field A. Camargo C. A. Taylor C. B. Barkey C. S. Roberts S. Colditz G. A. (2001). Peer, parent and media influences on the development of weight concerns and frequent dieting among preadolescent and adolescent girls and boys. *Pediatrics* 107 54–60. 10.1542/peds.107.1.54 11134434

[B17] Finne E. Bucksch J. Lampert T. Kolip P. (2011). Age, puberty, body dissatisfaction and psychical activity decline in adolescents. Results of German Health Interview and examination survey (KIGGS). *Int. J. Behav. Nutr. Phys. Act.* 8 119–133. 10.1186/1479-5868-8-119 22032266 PMC3231807

[B18] Fredrickson B. L. Roberts T. A. (1997). Objectification theory. *Psychol. Women Q.* 21 173–206. 10.1111/j.1471-6402.1997.tb00108.x

[B19] Gajtkowska M. (2013). Obraz własnego ciała wspoìłczesnej młodziez?y a kultura popularna. Badania własne [Self-image Body of Contemporary Youth and Pop Culture, Own research]. *Kult. Społecz. Edukacja* 2 103–118. 10.14746/kse.2013.4.2.07

[B20] Garner D. M. (2004). *EDI-3. Eating Disorders Inventory.* Lutz, FL: Psychological Assessment Resources, Inc.

[B21] Głogowska J. Zatorska A. (2016). Wizerunek ciała dziewcząt w wieku 8 – 9 lat [Body image of girls aged 8 – 9 years]. *Zest Nauk. WSKFiT* 11 29–34.

[B22] Grabe S. Ward L. Hyde J. (2008). The role of the media in body image concerns among women: a meta-analysis of experimental and correlational studies. *Psychol. Bull.* 134 460–476. 10.1037/0033-2909.134.3.460 18444705

[B23] Halliwell H. (2013). The impact of thin idealized media images on body satisfaction: does body appreciation protect women from negative effects? *Body Image* 10 509–514. 10.1016/j.bodyim.2013.07.004 23972728

[B24] Hayes S. Tantleff-Dunn S. (2010). Am I too fat to be a princess? Examining the effects of popular children’s media on young girls’ body image. *Br. J. Dev. Psychol.* 28(Pt 2), 413–426. 10.1348/026151009X424240 20481395

[B25] Izydorczyk B. (2014). *Postawy i Zachowania Wobec Własnego Ciała w Zaburzeniach Odżywiania [Attitudes and Behavior Towards One’s Own Body in Eating Disorders].* Warszawa: Wydawnictwo Naukowe PWN.

[B26] Izydorczyk B. (2015a). Psychological and socio-cultural risk factors for developing negative attitude and anti-health behavior toward the body in young women. *Polish Psychol. Bull.* 46 555–572. 10.1515/ppb-2015-0062

[B27] Izydorczyk B. (2015b). A psychological typology of females diagnosed with anorexia nervosa, bulimia nervosa or binge eating disorder. *Health Psychol. Rep.* 3 312–325. 10.5114/hpr.2015.55169

[B28] Izydorczyk B. Rybicka-Klimczyk A. (2009). Poznawcze aspekty obrazu ciała u kobiet a zaburzenia odżywiania. *[Cognitive aspects of women’s body image and eating disorders]*. *Endokrynol. Pol.* 60 287–294. 19753543

[B29] Jones D. C. Crawford J. K. (2006). The peer appearance culture during adolescence: gender and body mass variations. *J. Youth Adolesc.* 35 257–269. 10.1007/s10964-005-9006-5

[B30] Józefik B. (2014). *Kultura, Ciało, (nie) Jedzenie. Terapia. Perspektywa Narracyjno - Konstrukcjonistyczna w Zaburzeniach Odżywiania [Culture, body, (not) food. Therapy. Narrative-constructivist perspective on eating disorders].* Kraków: Wydawnictwo Uniwersytetu Jagiellońskiego.

[B31] Kilpela L. S. Becker C. B. Wesley N. Stewart T. (2015). Body Image in Adult Women: Moving Beyond the Younger Years. *Adv. Eat. Disord.* 3 144–164. 10.1080/21662630.2015.1012728 26052476 PMC4452130

[B32] Knauss C. Paxton S. J. Alsaker F. D. (2009). Validation of the German version of the sociocultural attitudes towards appearance questionnaire (SATAQ-G). *Body Image* 6 113–120. 10.1016/j.bodyim.2009.01.002 19244000

[B33] Lamb S. Peterson Z. (2012). Adolescent girls’ sexual empowerment: Two feminists explore the concept. *Sex Roles* 66 703–712. 10.1007/s11199-011-9995-3

[B34] Lawler M. Nixon E. (2011). Body dissatisfaction among adolescent boys and girls: the effects of body mass, peer appearance culture and internalization of appearance ideals. *J. Youth Adolesc.* 40 59–71. 10.1007/s10964-009-9500-2 20058058

[B35] Levine M. P. Murnen S. K. (2009). Everybody knows that mass media are/are not [pick one] a cause of eating disorders”: a critical review of evidence for a causal link between media, negative body image, and disordered eating in females. *J. Soc. Clin. Psychol.* 28 9–42. 10.1521/jscp.2009.28.1.9

[B36] Levine M. P. Smolak L. (1996). “Media as a context for the development of disordered eating,” in *The Developmental Psychopathology of Eating Disorders*, eds Smolak L. Levine M. P. Striegel-Moore R. (Mahwah, NJ: Erlbaum), 235–257.

[B37] Levine M. P. Smolak L. (2006). *The Prevention of Eating Problems and Eating Disorders: Theory, Research, and Practice.* Mahwah, NJ: Lawrence Erlbaum Associates.

[B38] Levine M. P. Smolak L. Hayden H. (1994). The relation of sociocultural factors to eating attitudes and behaviors among middle school girls. *J. Early Adolesc.* 14 471–490. 10.1177/0272431694014004004 8124323

[B39] Mäkinen M. Pukko-Viertomies L.-R. Lindberg N. Siimes M. A. Alberg V. (2012). Body dissatisfaction and body mass in girls and boys transitioning from early to mid adolescence: additional role of self-esteem and eating habits. *BMC Psychiatry* 12:35. 10.1186/1471-244X-12-35 22540528 PMC3370989

[B40] McCabe M. Ricciardelli L. A. (2004). A longitudinal study of pubertal timing and extreme body change behaviors among adolescent boys and girls. *Adolescence* 39 145–166. 15230071

[B41] McCabe M. Ricciardelli L. A. (2009). Extreme weight change behaviours: are overweight and normal weight adolescents different, and does this vary over time? *Eur. Eat. Disord. Rev.* 17 301–314. 10.1002/erv.929 19378347

[B42] McCabe M. P. Fuller-Tyszkiewicz M. Mellor D. Ricciardelli L. Skouteris H. Mussap A. (2012). Body satisfaction among adolescents in eight different countries. *J. Health Psychol.* 17 693–701. 10.1177/1359105311425274 22021271

[B43] McCabe M. P. Ricciardelli L. A. (2003). Sociocultural influences on body image and body changes among adolescent boys and girls. *J. Soc. Psychol.* 143 5–26. 10.1080/00224540309598428 12617344

[B44] Mellor D. McCabe M. Ricciardelli L. Yeow J. Daliza N. Hapidzal N. F. (2009). Sociocultural influences on body dissatisfaction and body change behaviors among Malaysian adolescents. *Body Image* 6 121–128. 10.1016/j.bodyim.2008.11.003 19195942

[B45] Menzel J. E. Sperry S. L. Small B. Thompson J. K. Sarwer D. B. Cash T. F. (2011). Internalization of appearance ideals and cosmetic surgery attitudes: a test of the tripartite influence model of body image. *Sex Roles* 65 469–477. 10.1007/s11199-011-9983-7

[B46] Mond J. Van den Berg P. Boutelle K. Hannan P. Neumark-Sztainer D. (2011). Obesity, body dissatisfaction, and emotional well-being in early and late adolescence: finding from the project EAT study. *J. Adolesc. Health* 48 373–378. 10.1016/j.jadohealth.2010.07.022 21402266 PMC3214691

[B47] Neumark-Sztainer D. Story M. Hannan P. J. Perry C. Irving L. M. (2002). Weight-related concerns and behaviors among overweight and non-overweight adolescents: implications for preventing weight-related disorders. *Arch. Pediatr. Adolesc. Med.* 156 171–178. 10.1001/archpedi.156.2.171 11814380

[B48] Nielson H. E. Reel J. J. Galli N. A. Crookston B. T. Miyairi M. (2013). Body image and westernization trends among Japanese adolescents. *Health Educ.* 45 4–10.

[B49] Petersson S. Johnsson P. Perseius K. I. (2017). A Sisyphean task: experiences of perfectionism in patients with eating disorders. *J. Eat. Disord.* 5:3. 10.1186/s40337-017-0136-4 28261478 PMC5327572

[B50] Rodgers R. Chabrol H. Paxton S. J. (2011). An exploration of the tripartite influence model of body dissatisfaction and disordered eating among Australian and French college women. *Body Image* 8 208–215. 10.1016/j.bodyim.2011.04.009 21664887

[B51] Rodgers R. F. (2016). The role of the “Healthy Weight” discourse in body image and eating concerns: an extension of sociocultural theory. *Eat. Behav.* 22 194–198. 10.1016/j.eatbeh.2016.06.004 27299698

[B52] Sánchez-Carracedo D. Barrada J. R. López-Guimerà G. Fauquet J. Almenara C. A. Trepat E. (2012). Analysis of the factor structure of the sociocultural attitudes towards appearance questionnaire (SATAQ-3) in Spanish secondary-school students through exploratory structural equation modeling. *Body Image* 8 208–215. 10.1016/j.bodyim.2011.10.002 22088493

[B53] Shroff H. Thompson J. K. (2006). The tripartite influence model of body image and eating disturbance: a replication with adolescent girls. *Body Image* 3 17–23. 10.1016/j.bodyim.2005.10.004 18089205

[B54] Stice E. Killen J. D. Hayward C. Taylor C. B. (1998). Support for the continuity hypothesis of bulimic pathology. *J. Consult. Clin. Psychol.* 66 784–790. 10.1037/0022-006X.66.5.784 9803697

[B55] Stice E. Marti C. N. Rohde P. (2013). Prevalence, incidence, impairment an course of the proposed DSM-V eating disorder diagnoses in 8-year prospective community study of young women. *J. Abnorom. Psychol.* 122 445–457. 10.1037/a0030679 23148784 PMC3980846

[B56] Stice E. Marti C. N. Spoor S. Presnell K. Shaw H. (2008). Dissonance and healthy weight eating disorder prevention programs: long-term effects from a randomized efficacy trial. *J. Consult. Clin. Psychol.* 76 329–340. 10.1037/0022-006X.76.2.329 18377128 PMC2677629

[B57] Stice E. Schupak-Neuberg E. Shaw H. E. Stein R. I. (1994). Relation of media exposure to eating disorder symptomatology: a examination of mediating mechanisms. *J. Abnorm. Psychol.* 103 836–840. 10.1037/0021-843X.103.4.836 7822589

[B58] Stice E. Whitenton K. (2002). Risk factors for body dissatisfaction in adolescent girls: a longitudinal investigation. *Dev. Psychol.* 38 669–678. 10.1037//0012-1649.38.5.669 12220046

[B59] Striegel-Moore R. H. Roselli F. Perrin N. DeBar L. Wilson G. T. Mag A. (2009). Gender diferrence in the prevalaence of eating disorder symptoms. *Int. J. Eat. Disord.* 42 471–474. 10.1002/eat.20625 19107833 PMC2696560

[B60] Swami V. Frederick D. A. Aavik T. Alcalay L. Allik J. Anderson D. (2010). The attractive female body weight and female body dissatisfaction in 26 countries across 10 world regions: results of the international body project I. *Pers. Soc. Psychol. Bull.* 36 309–325. 10.1177/0146167209359702 20179313

[B61] Thompson K. J. Van den Berg P. Roehrig M. Guarda A. S. Heinberg L. J. (2004). The socjocultural attitudes towards appearance Scale-3 (SATAQ-3). Development and Vaidation. *J. Eat. Disord.* 35 293–300. 10.1002/eat.10257 15048945

[B62] Thomsen S. R. Weber M. M. Brown L. B. (2002). The relationship between reading beauty and fashion magazines and the use of pathogenic dieting methods among adolescent females. *Adolescence* 37 1–18. 12003283

[B63] Tiggemann M. (2003). Media exposure, body dissatisfaction and disordered eating: television and magazines are not the same. *Eur. Eat. Disord. Rev.* 11 418–430. 10.1002/erv.502

[B64] Wilcox K. Laird J. D. (2000). The impact of media images of super-slender women and women’s self-esteem: Identification, social comparison, and self-perception. *J. Res. Pers.* 34 278–286. 10.1006/jrpe.1999.2281

[B65] Xu X. Mellor D. Kiehne M. Ricciardelli L. A. McCabe M. P. Xu Y. (2010). Body dissatisfaction, engagement in body change behaviors and sociocultural influences on body image among Chinese adolescents. *Body Image* 7 156–164. 10.1016/j.bodyim.2009.11.003 20089467

[B66] Żechowski C. (2008). Polska wersja Kwestionariusza Zaburzenì Odżywiania (EDI) – adaptacja i normalizacja [Polish Version of Eating Disorder Inventory – adaptation and normalization]. *Psychiatr. Pol.* 42 179–193. 19697524

